# Investigating the accuracy of mandibulectomy and reconstructive surgery using 3D customized implants and surgical guides in a rabbit model

**DOI:** 10.1186/s40902-023-00375-9

**Published:** 2023-01-26

**Authors:** Min Keun Kim, Min Ji Ham, Won Rae Kim, Hyung Giun Kim, Kwang Jun Kwon, Seong Gon Kim, Young Wook Park

**Affiliations:** 1 Department of Oral and Maxillofacial Surgery, College of Dentistry, Gangneung-Wonju National University, Gangneung, 25457 Republic of Korea; 2 Functional Materials and Components R&D Group, Korea Institute of Industrial Technology, Gangneung, 25440 Republic of Korea

**Keywords:** 3D printing, Customized titanium implant, Surgical guide, Accuracy, Rabbit model

## Abstract

**Background:**

This study aimed to analyze the accuracy of the output of three-dimensional (3D) customized surgical guides and titanium implants in a rabbit model, and of mandibulectomy, reconstructive surgery, and surgical outcome; additionally, the correlation between surgical accuracy and surgical outcomes, including the differences in surgical outcome according to surgical accuracy, was analyzed.

**Results:**

The output of implants was accurately implemented within the error range (− 0.03–0.03 mm), and the surgical accuracy varied depending on the measured area (range − 0.4–1.1 mm). Regarding surgical outcomes, angle between the mandibular lower borders showed the most sensitive results and distance between the lingual cusps of the first molars represented the most accurate outcomes. A significant correlation was noted between surgical accuracy in the anteroposterior length of the upper borders pre- and postoperatively and the angle between the mandibular lower borders (regression coefficient = 0.491, *p* = 0.028). In the group wherein surgery was performed more accurately, the angle between the mandibular lower borders was reproduced more accurately (*p* = 0.021). A selective laser melting machine accurately printed the implants as designed. Considering the positive correlation among surgical accuracy in the mandibular upper borders, angle between the mandibular lower borders, and more accurately reproduced angle between the mandibular lower borders, the angle between the mandibular lower borders is considered a good indicator for evaluating the outcomes of reconstructive surgery.

**Conclusion:**

To reduce errors in surgical outcomes, it is necessary to devise a positioner for the surgical guide and design a 3D surgical guide to constantly maintain the direction of bone resection. A fixed area considering the concept of three-point fixation should be selected for stable positioning of the implant; in some cases, bilateral cortical bone fixation should be considered. The angle between the mandibular lower borders is a sensitive indicator for evaluating the outcomes of reconstructive surgery.

## Background

The oral and facial areas are aesthetically and functionally important. The mandible is responsible for the shape of the lower facial skeleton and assists the primary digestion of food in mouth aided by mastication, thus playing a vital role in living a healthy life. Various types of bone defects can occur in the mandible because of large benign tumors, malignant tumors, jaw osteomyelitis, osteonecrosis, congenital anomalies, and severe trauma. In such cases, the defective areas should be reconstructed to restore the aesthetics of facial areas and function of the mandible [1].

Defects in the mandible can occur in extremely diverse and complex ways; therefore, numerous factors should be considered during its reconstruction, depending on the location or extent of the defect. In cases wherein continuity of the mandible is lost, restoration of the occlusal relationship between the maxillary and mandibular teeth should be primarily addressed for functional recovery. Therefore, both mandibular condyles must be maintained in a functionally balanced positional relationship and, simultaneously, the continuity of the mandible should be restored while maintaining the occlusal relationship between the maxillary and mandibular teeth. In addition, the morphological aesthetics of the lower facial area should be considered while planning and conducting reconstructive surgery [2]. In case of mandibular defects, including the mandibular condyle responsible for the mandible’s joint function, reconstructive surgery should be planned and conducted while focusing on the opening function of the mandible to ensure maintenance of smooth opening and closing of the mouth, thus restoring the mastication function [3]. Restoration poses further challenges when the entire mandible is defective. In such cases, along with the restoration of the lower facial area primarily via mandibular reconstruction, the opening, closing, and mastication functions of the mouth should be restored, which renders the procedure to be an extremely complex reconstructive surgery [4]. Therefore, for improving the quality of life of patients, it is important to aesthetically and functionally reconstruct various defective areas of the mandible, which is challenging for clinicians.

Reconstructive surgeries for mandibular defects have been conducted using fibula flaps or iliac flaps, accompanied by microvascular anastomosis [5, 6]. However, this method of mandibular reconstructive surgery has several limitations. First, the appearance of the mandible must be restored for aesthetic recovery of the lower facial area; however, the fibula is quite thin and if the mandibular lower edges are aligned during reconstructive surgery, a crown shape of considerable length is created in case of a dental implant surgery for occlusion. In such cases, the long-term prognosis is not favorable. Second, if the fibula is placed upward to improve the crown-to-root ratio, achieving the aesthetic formation of the lower facial area becomes difficult, thus posing another limitation. Considering these limitations, the double-barrel technique was implemented to increase the height of the fibula using two fibula layers. However, this causes complications in reconstructive surgery, posing a risk of potential flap failure if the vascular pedicle is pressed [7]. The iliac flap is an ideal mandible reconstruction method because it facilitates the maintenance of the crown-to-root ratio of a dental implant and the shape of the lower facial area. However, the deep circumferential iliac artery pedicle used for flap surgery is short, with a mean length of 4–7 cm and the mean diameter of the blood vessels used for microscopic surgery is 1.5–3 mm, which is relatively narrow compared with that of the fibula flap, rendering the surgery difficult and limiting its use [8]. In addition, in case of a defect affecting the entire mandible, planning an ideal reconstruction surgery for aesthetic and functional purposes is difficult because the amount of the donor area is quite limited to facilitate reconstruction using a fibula flap or an iliac flap. Elderly patients with debilitating conditions require surgery at the secondary donor site, and additional time is needed for microvascular surgery, thereby posing a challenge.

Various clinical trials have been conducted recently using three-dimensional (3D) printers that are helpful in overcoming the abovementioned limitations of reconstructive surgery. Examples of clinical applications of 3D printers include eye socket reconstruction and craniofacial plastic surgery as well as use in cases of complex maxillary defects [9–13]. Favorable results have been reported for spinal surgery in the field of orthopedics and sternal reconstruction in the field of thoracic surgery [14, 15]. This method has been used in cases of defective mandible reconstruction wherein the continuity of the mandible was lost and the mandible, including the mandibular joint and entire mandible [2–4], was affected. In addition, it has been used in plastic surgery for facial contouring [16]. In orthognathic surgery, bone resection is performed as per the bone resection guide produced by the 3D printer consistent with the surgery plan, and postoperatively, the 3D printer creates a board that fits into the maxillary and mandibular positions, thereby enabling the fixing of the metal plate immediately without the need for bending in the operating room, which reduces the surgery time and provides more predictable surgical outcomes [16–18].

In an ideal method for the reconstruction of complex and diverse bone defects, the bone defects are primarily evaluated using computed tomography (CT) scans on a computer and reconstructive surgery is planned. Next, a bone resection guide and an implant for use in the reconstructive surgery are designed. Finally, these are created using a 3D printer and applied to clinical cases. This method is being attempted in several clinical fields. With the accumulation of results of these clinical trials, reconstructive surgery of the maxillofacial area has evolved from methods completely dependent on conventional free vascularized bone grafts to those involving maxillofacial implants using a computer and 3D printing [5–8, 19, 20].

To accurately employ such 3D-printed implants in reconstructive surgery, it is essential to use a surgical guide from the stage of bone resection [21]. In addition, the location of the bone resection guide must be accurately reproduced in the operating room, thus a navigation surgery is occasionally performed [22]. However, navigation requires expensive equipment, which is indispensable while performing this surgery. Moreover, various types of surgical bone resection inducers have been designed and introduced by clinicians to provide a more accurate surgical guide [23, 24].

However, in reality, using such 3D outputs in reconstructive surgery has not been accepted as a general practice by clinicians. The reasons for this include clinicians’ anxiety regarding the accuracy of the output, uncertainty on whether the surgery will be correctly performed in the operating theater despite appropriate output, uncertainty regarding the initial and long-term clinical results, and financial burden on patients. Amid these concerns, accumulation of clinical results is required to determine the initial and long-term clinical experiences, and efforts should be made to reduce the financial burden on patients associated with an increase in demand owing to the increase in clinical cases. Therefore, in the present study, a mandibular bone resection model of rabbits was created and reconstructive surgery was performed using a 3D-printed surgical bone resection guide and implant. Pre- and postoperative data were used to analyze the accuracy of the 3D output and reconstructive surgery, and to determine the initial clinical results of the reconstructive surgery. In addition, this study aimed to analyze the correlation between the accuracy of surgery and clinical results and the variation of clinical results according to surgical accuracy.

## Methods

### Experimental animals

In the present study, 50-week-old male rabbits (New Zealand white rabbits, body weight 3–4 kg, *n* = 20) were used. The rabbits were reared for 40 weeks in a well-managed environment with a sufficient adjustment period. This study was approved by the XXXX Committee of XXXX, XX, XX (XXXX-XXXX-X).

### Preoperative CT of experimental animals

At 1-week preoperatively, all 20 experimental animals received general anesthesia before undergoing CT (Alphard 3030, ASAHI ROENTGEN®). For general anesthesia, a mixture of 0.5 mL of tiletamine–zolazepam (Zoletil, Bayer Korea, Seoul, Korea) and 0.5 mL of xylazine hydrochloride (Rompun, Bayer Korea, Seoul, Korea) was administered.

### Designing and printing of customized surgical bone resection guides and implants

The Gangneung branch of Korea Institute of Industrial Technology designed and printed surgical bone resection guides and implants using CT data and MIMICS 23.0 software (Materialize HQ Technologielaan, Leuven, Belgium). Considering that the size of the mandible of the rabbit may change in case of a long period until output, the period from CT scan to printing an implant was set to < 1 week. A selective laser melting (SLM) machine was used for printing.

#### Design

**Fig. 1:**
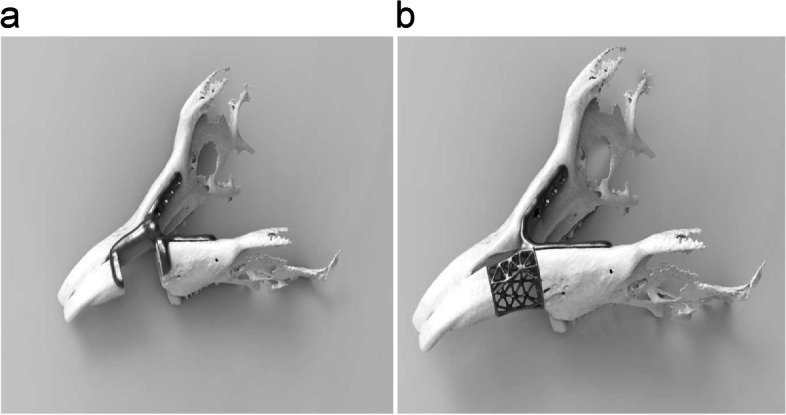
Design of bone resection guides and implants. **a** Bone resection guide; **b** implant

The customized mandibular resection guides and implants were designed using a software (3-matic, Materialize®) on a computer (Fig. 1). Individual CT data of rabbits were used for preparing the design. Customized bone resection guide and implant were created for each rabbit. A 10-mm defect was planned in the anterior part of the first mandibular molars, and the metal plate was designed to be lingual to the mandible for minimizing possible skin fistula formation. A fixing rod was designed for the four left and right sides. Microscrews (M3®, 1.2 mm) were used to fix the rod. To enhance connection to the screw head, aligning the shape of the screw hole to the screw head was attempted; however, it was not feasible using 3D printing. Therefore, it was manually performed. The bone defects were designed in the form of mesh to enable future bone grafts or dental implants. The thickness of the designed metal plate was 1 mm.

#### Output

The bone resection guides and implants were printed using an SLM machine (SLM 280). Commercialized pure titanium (CP-Ti, SLM solution®) was used for powder. The size of the spherical CP-Ti powder piece was 24.8–58.3 μm, and the mean size was 38.8 μm. The conditions of the printing process, including a laser power of 350 W, scan speed of 1400 mm/s, an interlayer thickness of 30 μm, oxygen pressure of 0.1%, and the temperature of the Ar inert gas and build plate at 200 °C were maintained as per the manufacturer’s instructions (SLM solution®).

### Surgery of experimental animals

**Fig. 2:**
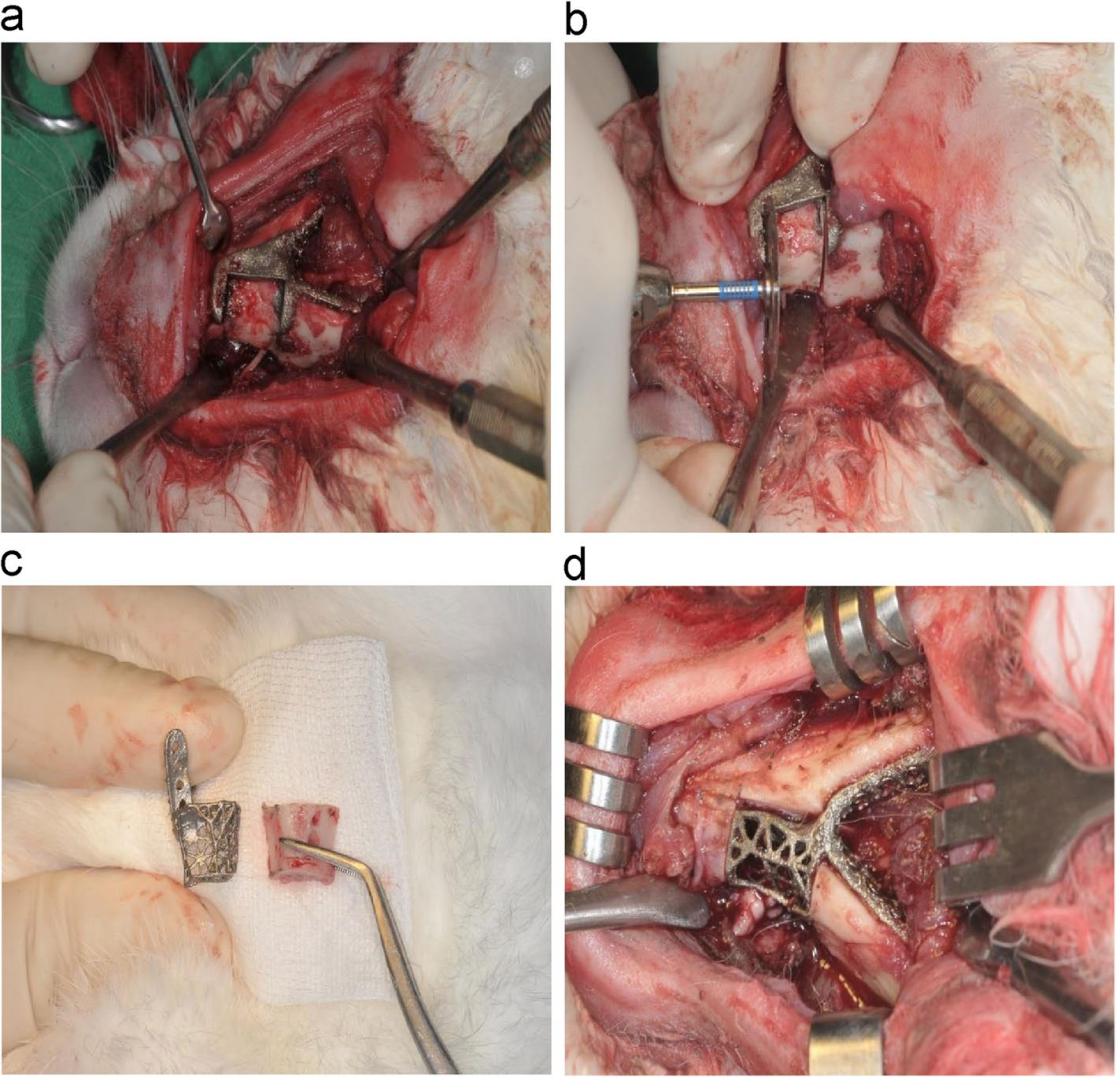
Animal surgery. **a** fixation of the bone resection guide and marking of the bone resection border (pencil); **b** bone resection using a disk; **c** resected bone fragment and printed implant; **d** positioning and fixation of the implant

Preoperatively, the occlusal state in the rabbits was recorded using a digital camera. A depilatory agent was completely applied for approximately 3 min for hair removal. The incision line was designed to avoid both submandibular glands. Following skin incision, the muscle and periosteum were incised and detached, thereby exposing the mandible. First, the bone resection guide was fixed at a predetermined position (Fig. 2a). The bone was cut using a disk, as per the fixed bone resection guide (Fig. 2b). The cut mandibular bone fragments were separately stored in 99% ethyl alcohol for accuracy analysis of the surgery (Fig. 2c). All the screws in the bone resection guide were removed, and the output implant was placed at that location for fixation (Fig. 2d).

### Sacrifice and fixation of experimental animals

Carbon monoxide gas was used to sacrifice the experimental animals on day 1 postoperatively. Following sacrifice, the maxillary and mandibular bones were fixed together in 4% formalin solution for analysis of the reconstruction results in the cadaver.

### Restoration of 3D images pre- and postoperatively of the rabbit and output of the mandible model

**Fig. 3:**
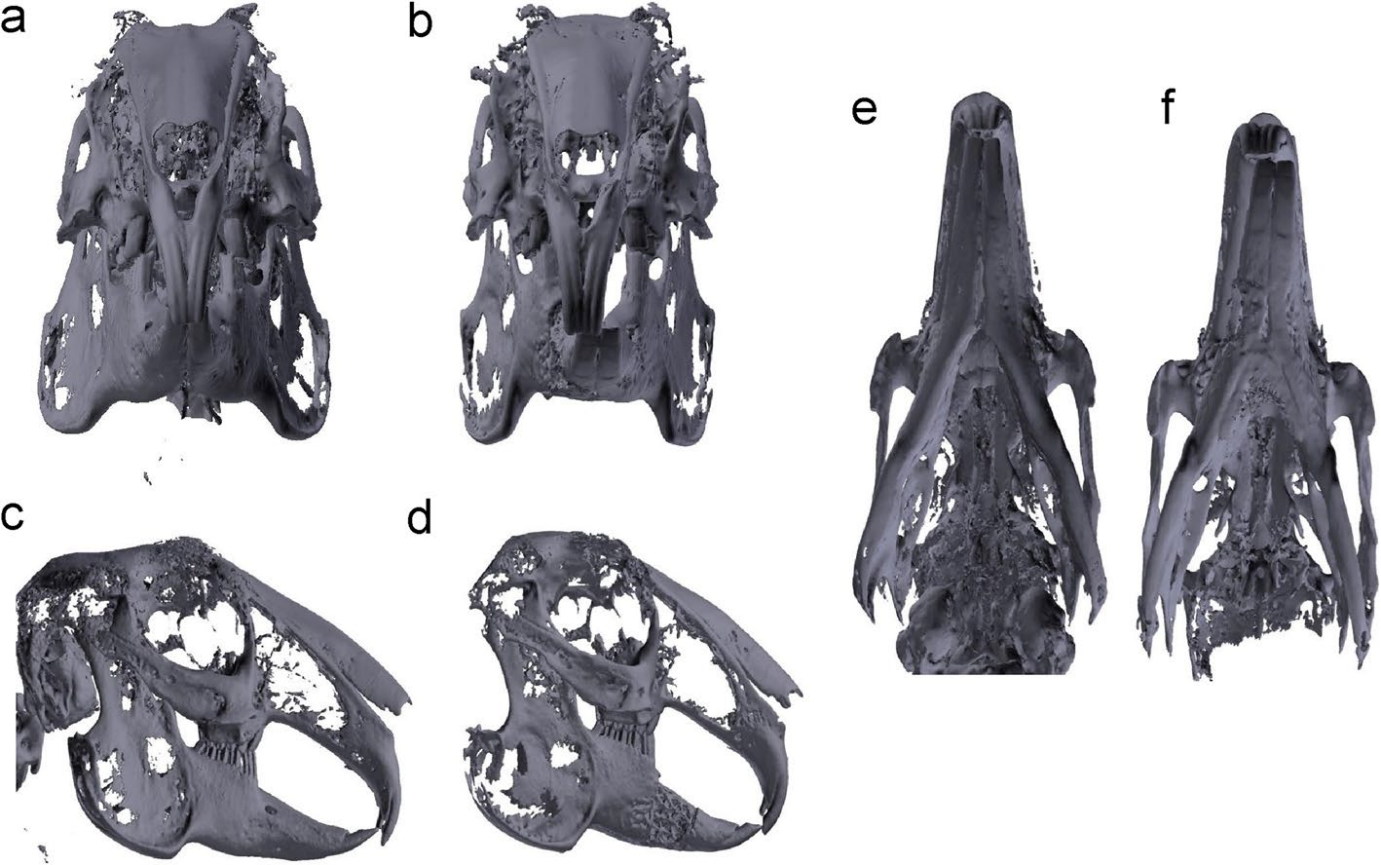
Three-dimensional (3D) reconstruction image of the mandible obtained pre- and postoperatively. **a** Frontal view preoperatively; **b** frontal view postoperatively; **c** lateral view preoperatively; **d** lateral view postoperatively; **e** basal view preoperatively; **f** basal view postoperatively

The 3D images were reconstructed using CT data of 20 rabbits pre- and postoperatively (Fig. 3a–f). In addition, all 3D models of 20 rabbits were printed and analyzed for determining the accuracy of mandibular reconstructive surgery. These 3D models were fabricated with the photosensitive polymer(VeroWhite) using the jetting type 3D printer by means of an Objet30 Prime (Stratasys).

### Selection and measurement of measuring points

#### Accuracy of printed implants and bone resection guides

1. Investigating the accuracy of the implants produced with regard to those specified in the surgery planning stage

The anteroposterior length of the implants in the mandibular lower border and that in the bone resection guide in the computer design were measured, and the lengths of the output implants and bone resection guide at the same site were measured and compared using vernier calipers .

2. Comparison of equivalence between planned and printed values

Three sections were considered (− 0.01–0.01, − 0.02–0.02, and − 0.03–0.03 mm) for equivalence comparison, and each section was analyzed to calculate the *p* value.

#### Surgical accuracy

**Fig. 4:**
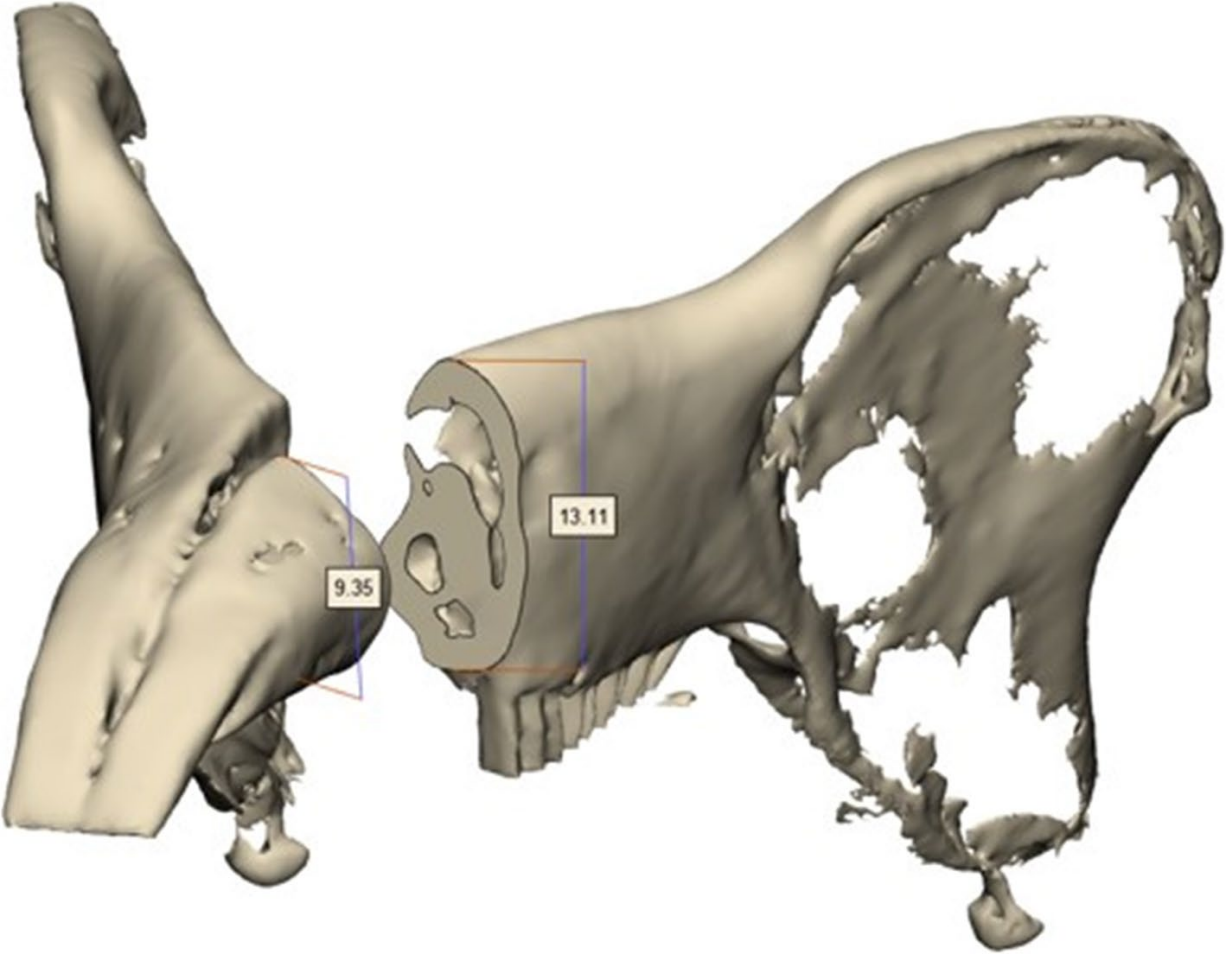
Measurement of the planned resection part length anterior height and posterior height and anteroposterior length of the upper and lower borders

**Fig. 5:**
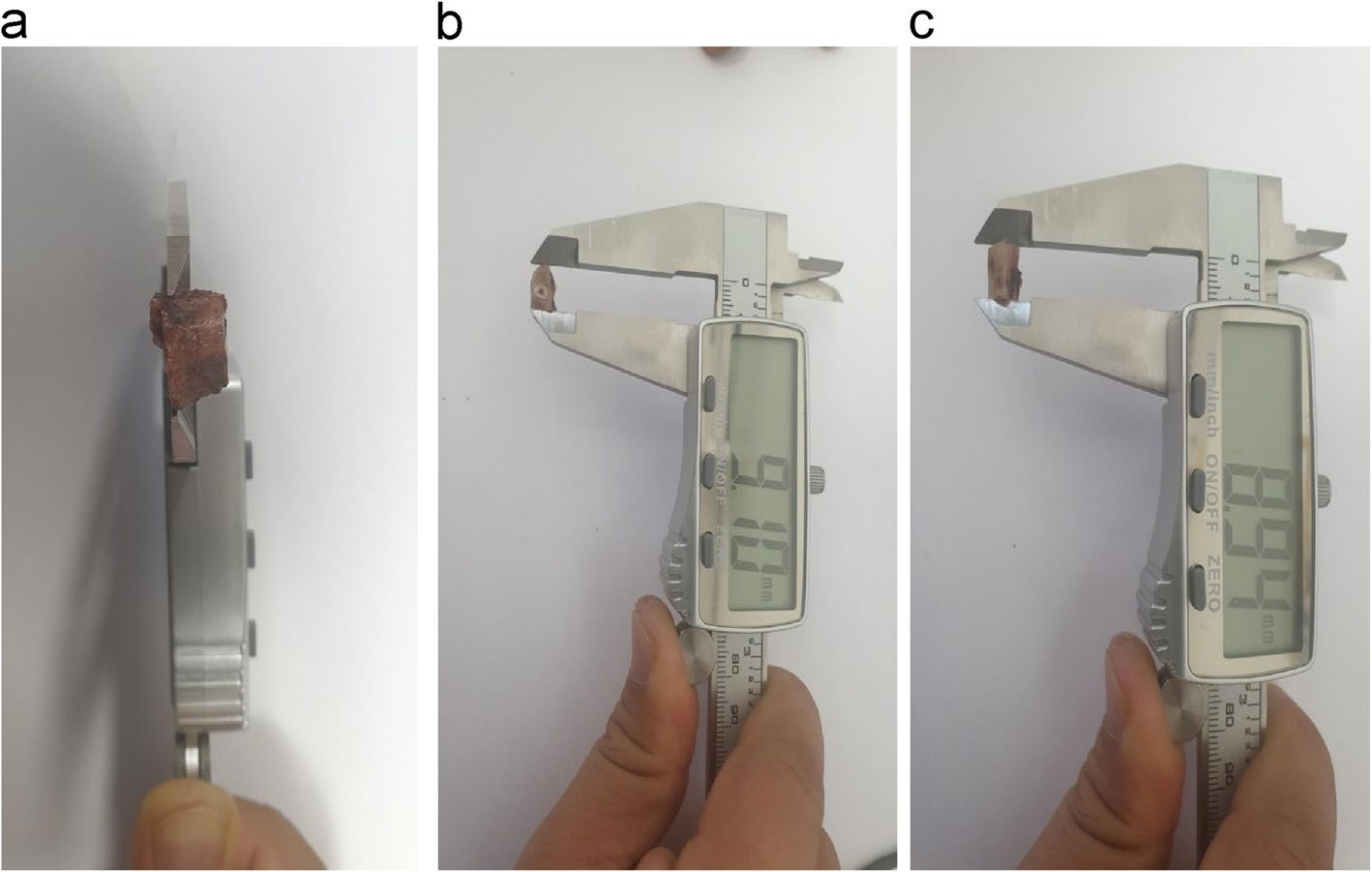
Measurement of the length of the actual resected bone. **a** Height of the posterior resection part; **b** height of the anterior resection part; **c** anteroposterior length of the lower border

1. To confirm whether the surgery was performed as planned for the planned length of bone resection, the anterior height, posterior height, and anteroposterior lengths at the mandibular upper and lower borders were measured in the computer design (Fig. 4). Further, the same parts were measured in the actually resected bone fragments using vernier calipers (Fig. 5). These two sets of values were compared.
2. For equivalence analysis between planned values and actual values, the entire distance was divided into six equivalence sections (− 0.3–0.3, − 0.4–0.4, − 0.5–0.5, − 0.6–0.6, − 1.0–1.0, and − 1.1–1.1 mm), and the *p* value was calculated for each section.

#### Assessment of surgical outcome

1. Assessment of dental midline

To confirm the changes in the dental midline, two measurements were compared: the position of the mandibular midline measured on the preoperative CT reconstruction image based on the midline of the maxillary central incisor and the position of the mandibular midline measured on the postoperative CT reconstruction image.

2. Assessment of the width of the mandible

**Fig. 6:**
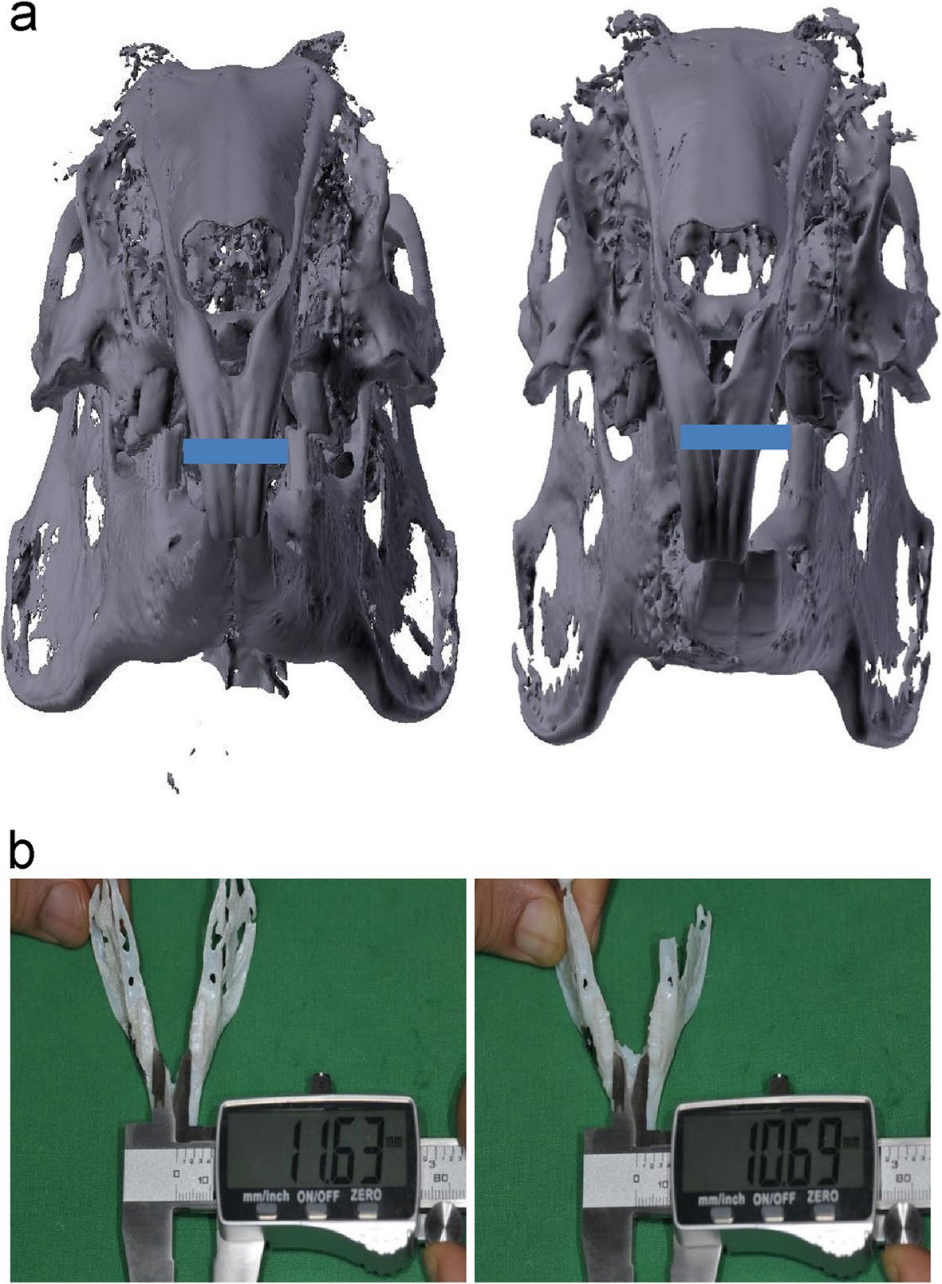
Measurement of the mandibular width. **a** The distance between the lingual cusps of the first mandibular molars in the three-dimensional (3D) output model constructed preoperatively (blue line); **b** the distance between the lingual cusps of the first mandibular molars in the 3D output model

To confirm the changes in the width of the mandible pre- and postoperatively, the distance between the lingual cusps of the first mandibular molars pre- and postoperatively were measured in the 3D output model and compared (Fig. 6).

3. Assessment of the occlusion

To confirm the changes of the occlusion, the distance between the cusps in the first maxillomandibular molars were measured. The distance between the cusps in the first maxillomandibular molars pre- and postoperatively was measured as 0 for complete bite, positive for open bite, and negative for scissors bite. The preoperative occlusal relationship was measured in anesthetized rabbit, and the postoperative occlusal relationship was measured in the cadaver model.

4. Assessment of the anterior mandibular length

To confirm the change in the anterior mandibular length, the distance from the mesial plane of the first mandibular molars to the incisal edge of the mandibular incisors were measured pre- and postoperatively in the 3D output model and compared.

5. Assessment of angles between mandibular lower borders

**Fig. 7:**
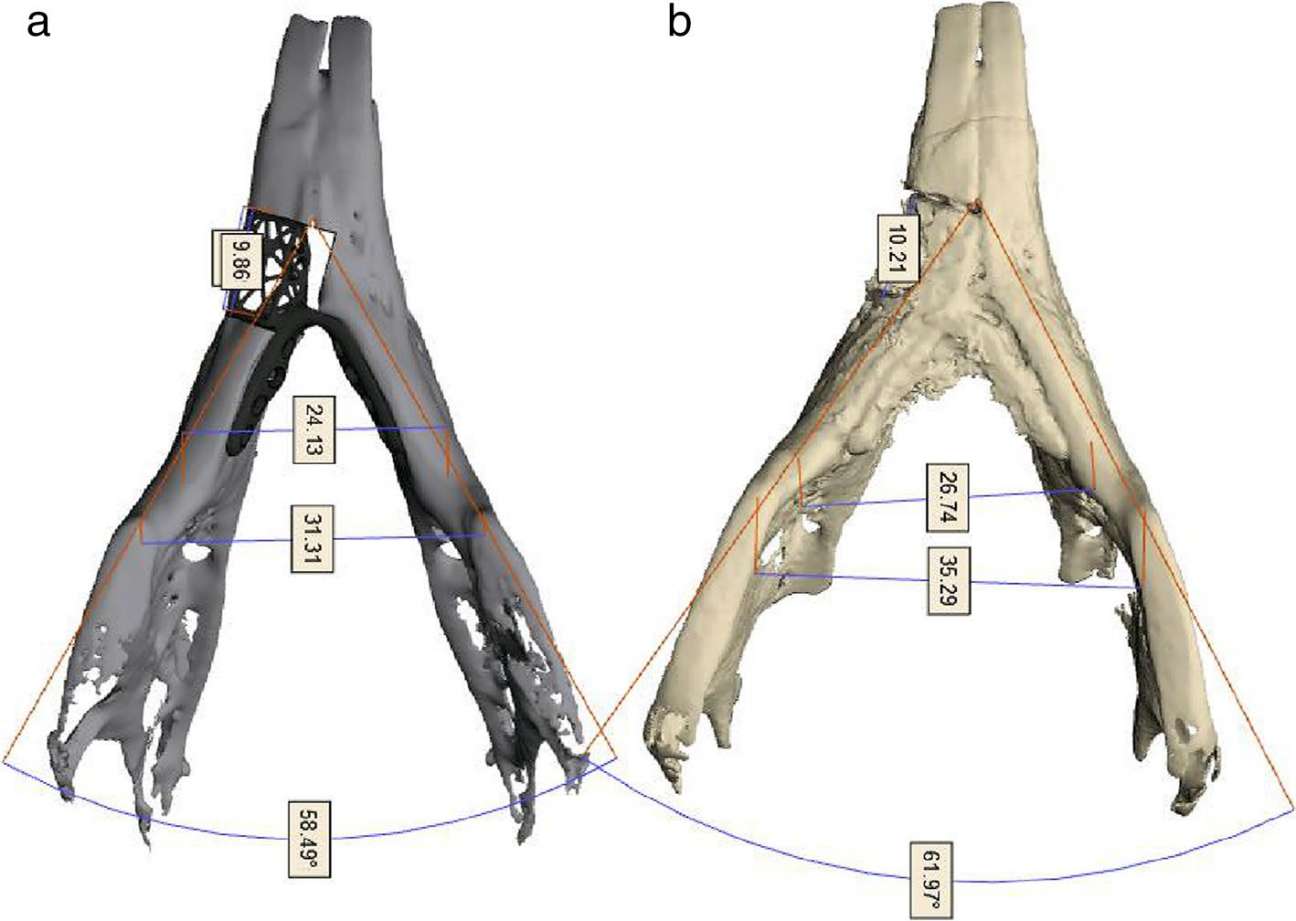
Measurement of the angle between mandibular lower borders. **a** Angle between mandibular lower borders in the three-dimensional (3D) reconstruction image obtained preoperatively; **b** angle between mandibular lower borders in the 3D reconstruction image obtained postoperatively

The angles between mandibular lower borders were measured on the 3D reconstructed images obtained from pre- and postoperative CT data and compared (Fig. 7).

6. Assessment of accuracy in surgical outcome

To analyze the accuracy of the surgical outcome for equivalence analysis of values pre- and postoperatively, the entire distance was divided into six equivalence sections (− 0.1–0.1 mm, − 0.5–0.5 mm, − 1.0–1.0 mm, − 2.0–2.0 mm, − 3.0–3.0 mm, and − 4.0–4.0 mm), and the *p* value was calculated for each section.

#### Assessment of the correlation between surgical accuracy and surgical outcome

Pearson correlation analysis was used to calculate the relationship between the surgical accuracy and various surgical outcomes, which was further tested for statistical significance.

#### Grouping according to the surgical outcome and assessment of differences in clinical outcomes between groups

When the difference in surgical outcome was < 0.25 mm or > 0.25 mm, it was categorized into group A or group B, respectively. The two groups were compared in terms of surgical outcomes.

### Statistical analysis

A paired sample *t* test was used to assess the accuracy of the output, performance, and surgical outcome of implant. Pearson correlation was used to analyze the correlation between surgical accuracy and surgical outcomes. In addition, an independent two sample *t*-test was used to determine the difference in surgical outcomes between groups according to the differences in surgical results. The software used for the analysis includes R Language ver. 3.3.3 (R Foundation for Statistical Computing, Vienna, Austria) and T&F software ver. 3.0 (YooJin BioSoft, Korea).

## Results

### Accuracy of the printed implants and bone resection guides

#### Planned and actual lengths (Table 1)

##### Results of equivalence comparison

In the − 0.03–0.03 mm equivalence section, the implant (*p* = 0.004) and bone resection guide (*p* = 0.012) were found to be equivalent (Table 2).

**Table 1:** Accuracy of printed implants and surgical bone resection guides

	Pre- and postoperative implants (mm)		Pre- and postoperative surgical bone resection guides (mm)	
	Planned	Output	Planned	Output
1	10.00	10.01	10.10	10.05
2	10.00	10.02	10.10	10.08
3	10.00	10.04	10.10	10.12
4	10.00	10.02	10.10	10.07
5	10.00	10.03	10.10	10.09
6	10.00	10.02	10.10	10.09
7	10.00	10.00	10.10	10.09
8	10.00	10.01	10.10	10.11
9	10.00	10.00	10.10	10.19
10	10.00	10.04	10.10	10.19
11	10.00	10.00	10.10	10.11
12	10.00	10.05	10.10	10.08
13	10.00	10.05	10.10	10.11
14	10.00	10.02	10.10	10.14
15	10.00	10.02	10.10	10.14
16	10.00	10.00	10.10	10.15
17	10.00	10.01	10.10	10.05
18	10.00	10.02	10.10	10.06
19	10.00	10.02	10.10	10.12
20	10.00	10.01	10.10	10.12
Average	10.00	10.02	10.10	10.11
SD	0.00	0.02	0.00	0.04

**Table 2:** Accuracy of printed implants and surgical bone resection guides: equivalence comparison

Variable	Mean	SE	95%CIs	*P* value (Equi. interval: − 0.01–0.01)	*P* value (Equi. interval: − 0.02–0.02)	*P* value (Equi. interval: − 0.03–0.03)
Pre- and postoperative implant: planned	10	0	10–10			
Pre- and postoperative implant: output	10.019	0.004	10.013–10.026			
Difference between pre- and postoperative findings: output–planned	0.019	0.004	0.013–0.026	0.993	0.444	0.004
Pre- and postoperative surgical bone resection guides: planned	10.1	0	10.1–10.1			
Pre- and postoperative surgical bone resection guides: output	10.108	0.009	10.09–10.126			
Difference in pre- and postoperative surgical bone resection guides: output−planned	0.008	0.009	− 0.01–0.026	0.413	0.099	0.012

*CI* confidence interval, *SE* standard error

### Accuracy in surgery

#### Lengths of planned and resected bone fragments (Table 3)

##### Results of equivalence comparison

The anteroposterior length at the mandibular upper border, height of the cut fragments in the posterior center, anteroposterior length at the mandibular lower border, and height of the cut fragments in the anterior center were equivalent in the − 0.4–0.4, − 0.5–0.5, − 0.6–0.6, and − 1.1–1.1 mm equivalence sections, respectively.

**Table 3:** Surgical accuracy

	Anterior bone height (mm)		Posterior bone height (mm)		Anteroposterior bone length (upper border) (mm)		Anteroposterior bone length (lower border) (mm)	
	Planned	Actual	Planned	Actual	Planned	Actual	Planned	Actual
1	9.35	10.59	13.11	13.37	10.00	9.14	10.00	9.04
2	10.43	10.47	13.52	13.37	10.00	9.78	10.00	9.7
3	9.68	10.01	13.57	12.59	10.00	9.28	10.00	9.8
4	9.06	9.22	12.28	11.31	10.00	9.18	10.00	9.48
5	10.61	10.66	14.09	13.08	10.00	10.13	10.00	9.51
6	10.00	10.78	13.72	13.42	10.00	10.39	10.00	9.86
7	9.46	10.48	13.33	12.99	10.00	9.97	10.00	9.73
8	9.10	9.82	13.43	13.06	10.00	10.5	10.00	9.17
9	8.33	8.98	11.44	11.35	10.00	10.96	10.00	9.87
10	9.81	10.04	13.86	13.48	10.00	10	10.00	9.84
11	8.89	9.83	12.30	12.11	10.00	9.72	10.00	9.67
12	8.88	9.51	11.61	11.41	10.00	9.1	10.00	9.25
13	8.78	9.67	12.70	12.74	10.00	9.94	10.00	9.76
14	9.17	10.31	13.19	13.42	10.00	9.57	10.00	9.65
15	8.25	10.38	11.63	12.83	10.00	10.01	10.00	9.78
16	9.54	11.13	13.35	14.19	10.00	10.75	10.00	10.3
17	9.41	9.78	12.26	13.32	10.00	9.63	10.00	9.7
18	8.15	9.73	12.46	12.11	10.00	10.59	10.00	10.01
19	8.60	9.73	11.84	12.33	10.00	10.12	10.00	10.88
20	8.67	9.32	12.40	11.72	10.00	10.18	10.00	9.55
Mean	9.21	10.02	12.80	12.71	10.00	9.974	10.00	9.7275
SD	0.68	0.57	0.82	0.82	0	0.56	0	0.39

*SD* standard deviation

### Assessment of surgical outcome

#### Assessment of dental midline

Postoperatively, all mandibles were displaced to the left, with a mean of 2.08 mm (range 0.01–6.95 mm).

#### Assessment of the width of the mandible

The mean distance between the lingual cusps of the first mandibular molars was 11.28 and 11.25 mm pre- and postoperatively, respectively—a reduction of 0.03 mm. In the comparison of surgical outcomes, this distance showed the most accuracy.

#### Assessment of the occusion

Out of a total of 20 cases, there were 6 cases of open bite on the left, 2 cases of open bite on the right, 5 cases of scissors bite on the right, and 7 cases of no open or scissors bite.

#### Assessment of the anterior mandibular length

The mean distance from the mesial plane of the first mandibular molars to the incisal edge of the incisors was 28.32 and 29.29 mm pre- and postoperatively, respectively—an increase of 0.97 mm.

#### Assessment of angles between mandibular borders

Angles between mandibular edges were 54.46° and 55.07° pre- and postoperatively, respectively—an increase of 0.61°.

#### Results of equivalence comparison for each equivalence section for the differences in values pre- and postoperatively (Table 4)

The distances between the lingual cusps of the first mandibular molars were equivalent in the − 0.5–0.5 mm equivalence section, showing the most accurate outcomes (*p* = 0.09). The variables that also showed equivalence include occlusion in the − 0.1–0.1 mm section (*p* = 0.09), distance from the mesial plane to the incisal edge of the incisors in the − 0.2–0.2 mm section (*p* < 0.01), angle difference between the mandibular edges in the − 0.03–0.03 mm section (*p* = 0.026), and the midline in the − 4.0–4.0 mm section (*p* = 0.03).

**Table 4:** Accuracy of surgical outcomes: equivalence comparison

Variable	Mean	SE	95%CIs	*P* value [1]	*P* value [2]	*P* value [3]	*P* value [4]	*P* value [5]	*P* value [6]
Dental midline: preoperative	0	0	0–0						
Dental midline: postoperative	2.803	0.391	2.037–3.569						
Difference in dental midline: postoperative–preoperative	2.803	0.391	2.037–3.569	1	1	1	0.973	0.31	0.003
Width of the mandible: preoperative	11.282	0.096	11.095–11.469						
Width of the mandible: postoperative	11.248	0.153	10.948–11.547						
Difference in width of the mandible: postoperative–preoperative	− 0.034	0.179	− 0.385–0.316	0.359	0.009	< 0.001	< 0.001	< 0.001	< 0.001
Occlusion: preoperative	0	0	0–0						
Occlusion: postoperative	1.380	0.309	− 0.409–0.801						
Difference in occlusion: postoperative–preoperative	1.380	0.309	− 0.409–0.801	0.62	0.168	0.009	< 0.001	< 0.001	< 0.001
Anterior mandibular length: preoperative	28.325	0.239	27.857–28.792						
Anterio mandibulr lenght: postoperative	29.291	0.238	28.824–29.758						
Difference in anterior mandibulr length: postoperative – preoperative	0.966	0.253	0.471–1.462	0.999	0.96	0.448	< 0.001	< 0.001	< 0.001
Angle between mandibular borders (°): preoperative	4.539	1.015	52.471–56.449						
Angle between mandibular borders (°): postoperative	5.121	1.145	52.745–57.235						
Difference in angle between mandibular borders (°): postoperative−preoperative	4.798	1.073	− 1.573–2.633	0.653	0.511	0.333	0.093	0.016	0.002

Width of the mandible: distance between the lingual cusps of the first mandibular molars

Occlusion: distance between the maxillomandibular first molars

Anterior mandibular length: distance between the mesial plane of the first mandibular molars to the incisal edge of the mandibular incisors

*CI* confidence interval, *SE* standard error

### Comparison of accuracy in surgical outcome (Fig. 8)

The most accurate surgical outcome was the distance between the lingual cusps of the first mandibular molars. The highest individual differences were observed in the angle between the mandibular lower borders.

**Fig. 8:**
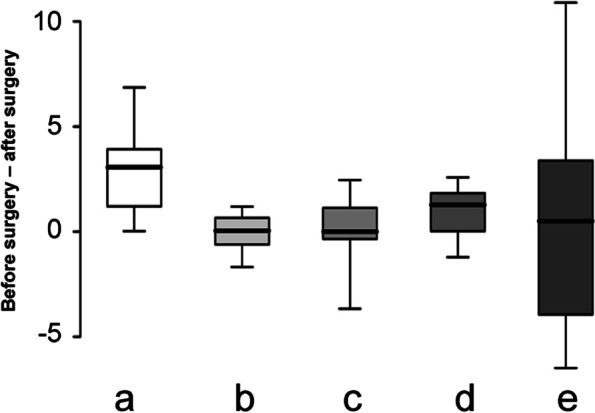
Box plot of various surgical outcomes pre- and postoperatively. **a** dental midline; **b** mandibular width **c** occlusion, **d** anterior mandibular length, **e** angle between mandibular lower borders

### Assessment of correlation between surgical accuracy and surgical outcome

**Fig. 9:**
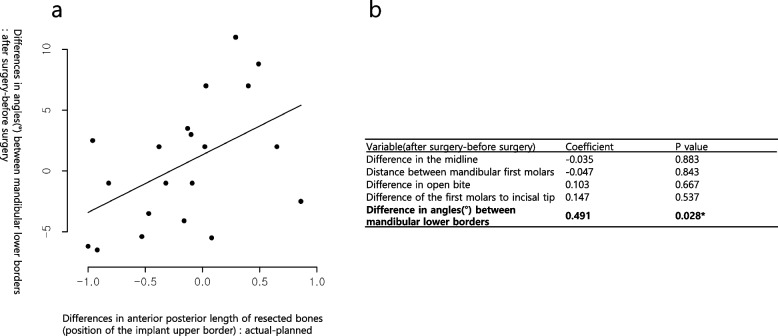
Analysis of correlation between pre- and postoperative length differences. Analysis of correlation between length differences in anteroposterior lengths of resected bones (upper borders) and angle differences in mandibular lower borders pre- and postoperatively

Regarding the correlation between surgical accuracy and surgical outcomes, a significant positive correlation was observed between surgical accuracy in the anteroposterior length of the upper border and the angle between the mandibular borders (regression coefficient = 0.491, *p* = 0.028; Fig. 9). No significant correlation was noted for other relationships between surgical accuracy and surgical outcomes.

### Grouping according to surgical outcome and assessment of differences in clinical outcomes between the groups

There was a significant difference in the angle between the mandibular borders (pre- and postoperative values) among groups A and B (*p* = 0.021). No significant results were observed in the correlation between surgical accuracy and surgical outcome.

## Discussion

Recently, several attempts have been made to use a 3D printer for surgery [25–30] and this method is applied to mandibular reconstruction. Reportedly, faster and more accurate surgical outcomes have been obtained using this method than those using previous reconstruction methods [31–36]. Moreover, 3D technology is currently applied to surgeries such as mandibular full arch reconstruction, which was previously difficult to perform without microscopic surgery [37]. Nevertheless, the technology using 3D printers is not widely used in clinical practice. This may be owing to a lack of confidence; lack of reliable data in terms of the accuracy of the 3D output; and concerns including whether surgery can be conducted as planned, whether desired clinical results can be obtained, and whether sufficiently stable results can be expected in the long term. Therefore, in the present study, a model of mandibular defect using 20 50-week-old male rabbits was constructed, reconstructive surgery using a 3D printer was conducted, pre- and postoperative data were analyzed, and the results were summarized. Furthermore, possible causes of error and methods for reducing it were considered. These results are expected to be used as reference for clinicians who intend to use 3D printers in clinical practice.

Regarding the evaluation of the accuracy of the output, the 3D printer used in this study was found to be accurate with an error range of − 0.03–0.03 mm (Table 1). Since the SLM machine used in this study can produce a mesh that is quite thin and facilitates the use of a dental implant drill, bone defects in this experiment were created in a mesh shape to ensure that they can be used in future bone transplantation and dental implants. The printed mesh-shaped bone defects were confirmed to be accurate with an error range of − 0.03–0.03 mm.

In surgical accuracy analysis, the mandibular upper edge was determined to be the most accurate and remained the same in the − 0.4–0.4 mm equivalence section. The mandibular lower edge was equivalent in the − 0.6–0.6 mm equivalence section. A more sophisticated surgery was performed in the mandibular upper edge because of two reasons. First, the bone resection guide was not designed to guide the bone resection direction in one direction, resulting in bone resection that was not uniformly unidirectional. Second, the disk used in the study was considerably large and while cutting the upper border, a slight change in direction led to additional cuts in parts of the lower border. In reality, the amount of bone removal at the lower border was higher by 0.247 mm than at the upper border. The bone resection guide was short; therefore, the bone resection required a marking with a pencil line, which was a contributing factor to the substantial error observed. These results might have been caused by the fact that as bone resection, starting at the lower border, proceeded to the upper border, even a slight change in the direction of bone resection led to further bone removal in the lower border. To overcome this challenge, the bone resection guide should be designed and manufactured to constantly maintain the direction of bone resection and guide bone resection on the entire resection site [21, 38–41]. In addition, it appears that an appropriate bone resection tool that fits into the resection site and guide might enhance surgical accuracy.

**Fig. 10:**
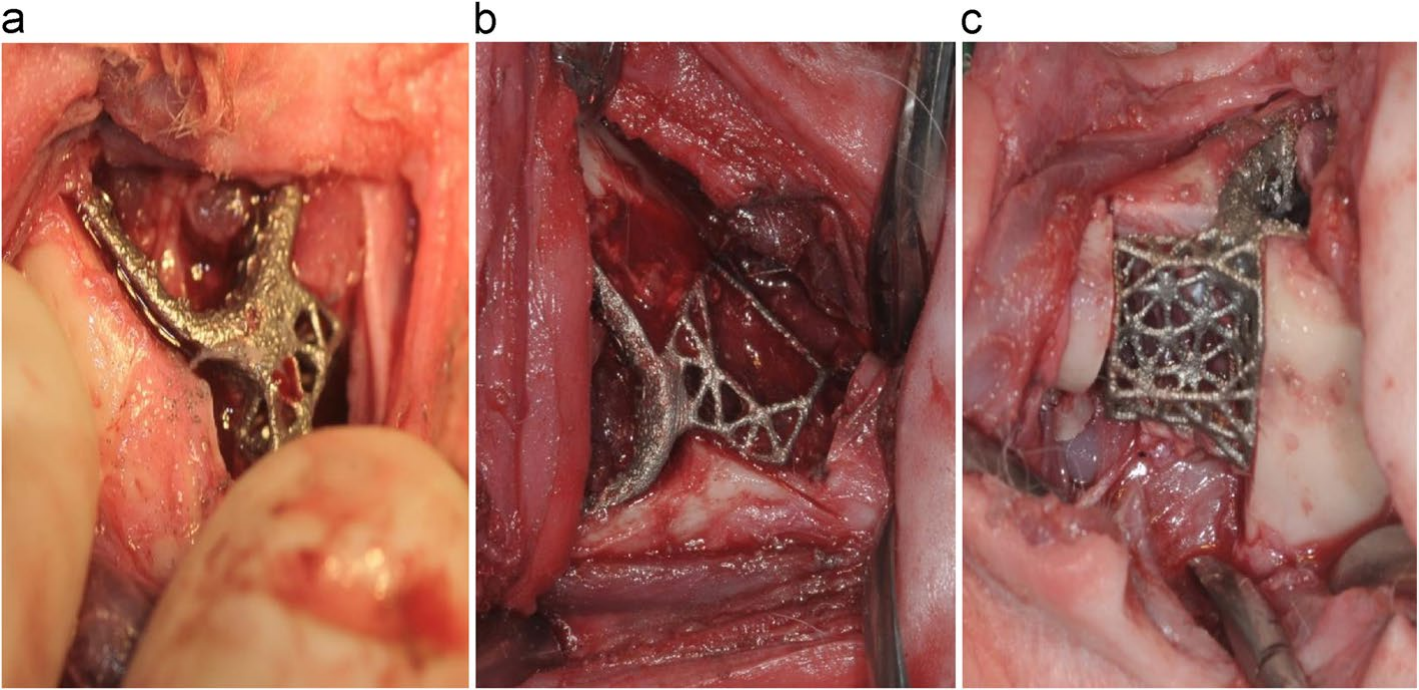
Cause of error during surgery. **a** Due to incorrect bone resection, interosseous interference occurred, which made it difficult to position the implant. **b** Due to incorrect positioning of the bone resection guide, the implant was mispositioned, creating a large space in the anterior part. **c** Due to incorrect angle of the bone resection, a space was created in the anterior part

According to the findings of surgical accuracy analysis, the height of the cut fragments in the posterior center was equivalent in the − 0.5–0.5 mm equivalence section and that of those in the anterior center was equivalent in the − 1.1–1.1 mm equivalence section. This substantial difference in the height of the anterior and posterior cut bone fragments appears to be an error wherein the bone resection guide was incorrectly positioned. However, checking whether the bone resection guide was properly positioned during the surgery was not feasible; therefore, surgery relied on the surgeon’s expertise, which was a major factor in prolonging the surgery. In addition, there were cases wherein the bone resection guide was incorrectly positioned and was recognized during the surgery. When the bone resection position was changed owing to the incorrect positioning of the bone resection guide, an interference occurred between the bone and implant, resulting in a displaced mandible (Fig. 10a). The accuracy of the height of the incisal bone in the posterior part was greater than that observed in the anterior part because the posterior incisal part includes an anatomical landmark located immediately in front of the mandibular molars, rendering the positioning easier during the surgery. To reduce the error due to the position of the guide while performing the surgery, using an adjacent anatomical landmark having an accurate position that does not move with the teeth would be a good approach. Therefore, it may be helpful to devise a bone resection guide positioner that facilitates fitting of the bone resection guide to the planned position [42–44]. Further extension of the bone resection guide so that it settles onto a certain anatomical structure, such as a tooth, is a good alternative approach to ensure easy positioning [45–47]. Navigation surgery is another way to check the position of the bone resection guide, but the equipment is expensive [48, 49].

The error in the 3D-printed materials was − 0.03–0.03 mm, but the error in the surgery was considerably high at − 0.4–1.1 mm. Efforts should be made to reduce these errors during the surgery. The following suggestions can be considered to reduce the errors during the surgery: first, a method to position the bone resection guide at the planned location during surgery (bone resection guide positioner) should be devised; second, it should be designed three dimensionally to maintain the direction of bone resection.

In surgical outcomes, the midline was displaced to the left in all 20 rabbits with a mean displacement of 2.8 mm. This is attributed to several reasons: first, presence of an interosseous interference due to an incorrect bone resection position (Fig. 10a); second, presence of an excessive interosseous gap due to an incorrect bone resection position (Fig. 10b); and third, loosening of the fixation or occurrence of a fracture in the fixed part of the mandibular lower edge, causing displacement. In such cases, the error in the bone resection position can be enhanced by designing an osteotome positioner, and the error due to loosened fixation can be improved by using stronger fixation such as bilateral cortical bone fixation. The distance between the lingual cusps of the first mandibular molars was relatively accurately reproduced when the occlusal relationship was well maintained. In case of open bite or scissors bite, there was a change in distance. Occlusion occurred in 6 cases of open bite on the left side and 5 cases of scissors bite on the right side. There were 2 cases of open bite on the right side and 7 cases of good occlusion. It appears that the open bite on the left and the scissors bite on the right is the displacement of the mandible caused by a muscle strain. These changes in occlusion appeared to have occurred because of loosened fixation and failure to perform intermaxillary fixation. During clinical practice, if surgery is accompanied by intermaxillary fixation and sufficiently strong fixation, considerably better outcomes can be expected.

The mean distance from the mesial plane of the first molars to the incisal edge was 0.97 mm. This was because the surgery does not exactly fit according to the original plan at the upper or lower borders, and there is a space between the osteotomizes, contributing to an increase in the distance. Since it was caused by failing to achieve bone resection as planned, it appears that the position of the osteotome can be accurately determined and the bone resection will be performed as planned, resulting in more accurate results. The angle between the mandibular lower edges increased by 0.61° from a mean of 54.46° preoperatively to 55.07° postoperatively. This region seems to be the position of the anterior part of the implant that exerted the greatest impact on the angle between the mandibular lower borders. Another reason for the large error observed in the present study was that the anterior part of the bone resection was not fixed. This deteriorated the stability of the anterior part of the implant. When the anterior part of the implant moves inward, the lower border angle increases, and when it moves outward, the lower border angle decreases, which affected reconstruction outcomes. Considering these outcomes, the fixed position of the implant should be selected based on the concept of at least a three-point fixation and the position of fixation should be determined by choosing points where the implant may not move or rotate, thereby minimizing the errors caused by implant movement.

Upon analyzing the correlation between various surgical outcomes and surgical accuracy, a positive correlation was observed between surgical accuracy of the mandibular upper border and the angle between the mandibular lower borders (Fig. 9). Based on these findings, the animals were categorized into two groups: one with accurate surgery and the other with inaccurate surgery; both groups were compared against the difference in angle between mandibular lower borders. When the surgery was performed relatively accurately, the difference in angle between the mandibular lower borders was small (Table 5). With relatively accurate surgery, it was easier to obtain positional stability even without fixation in front of the implant, resulting in better reproduction of the angle between the mandibular lower borders. The angle between the mandibular lower borders varied according to individual animals (Fig. 8) and was the most sensitive index among various surgical outcomes. Furthermore, it was significantly correlated with surgical accuracy (Fig. 9). This appears to be useful in assessing the accuracy of mandibular reconstruction in the future.

**Table 5:** A comparison of the differences in angles between the mandibular edges of both groups

Variable	Group A: < − 0.115 (*N* = 10, 50%)	Group B: ≥ − 0.115 (*N* = 10, 50%)	*p* value
Differences in angles between the mandibular borders: After–before surgery	− 1.97 ± 1.18	3.18 ± 1.67	0.021^*^

The table shows a comparison of the differences in angles between the mandibular borders of both groups according to the differences of surgical outcomes at the mandibular upper borders

Considering the procedures conducted in the present study, surgical outcomes were assessed using postoperative CT scans, the accuracy of the operation was assessed, and the causes of error were analyzed. These procedures can provide basic data for reducing errors in reconstructive surgery using 3D printing enabling more stable surgical outcomes. Therefore, clinicians should continue to make efforts to obtain a CT scan postoperatively to confirm the surgical outcome and analyze the causes of error. In addition, they should develop more stable indicators for assessing surgical outcomes and conduct follow-up observations to increase the accuracy of reconstructive surgery using 3D outputs.

Further research is required in the several areas. First, the most important factor in performing an accurate surgery is positioning of the printed bone resection guide and implant as planned. Therefore, studies should be conducted to develop a positioner based on an anatomically stable position. Second, to reduce causes of the error, a 3D surgical bone resection guide should be developed, which is sufficient to reproduce the bone resection angle, and a corresponding osteotome should be prepared. Third, the location of fixation should be selected using the concept of three-point fixation to sufficiently maintain the position of the implant, and if necessary, sufficiently strong fixation via bilateral cortical bone fixation should be obtained. Fourth, in clinical practice, intermaxillary fixation should be used extensively to get an additional fixation effect and maintain the occlusal relationship. Among these future research approaches, development of a bone resection guide positioner is considered the most important factor for performing surgery, since accurate surgical performance can only be expected when such a positioner becomes available. It is necessary to develop a positioner based on a stable structure that does not change in position (e.g., dentition) and apply it during surgery. In addition, it is vital to sufficiently incise and detach during surgery for securing a sufficient field of view that is required when inserting an implant. When the bone resection guide was not positioned at the desired position, interosseous interference occurred, resulting in an undesired surgical outcome. Efforts are required to design an implant to reduce the possibility of interference.

Methods used in this study included carcass analysis of the experimental animals, analysis of cut mandible fragments, assessment of printed 3D models of rabbits constructed pre- and postoperatively, analysis of 3D images reconstructed on the computer, assessment of the surgical bone resection guide used in the surgery, and assessment of the clinical photos obtained pre- and perioperatively. To assess the prognosis of reconstruction surgery and the need for a revision surgery, the procedures for assessing surgical outcomes using 3D reconstruction images that can be applied in clinical settings, obtained pre- and postoperatively, and analyzing the cause of error will be helpful. In addition, it appeared that among the various indicators used to assess surgical outcomes in this study, the distance between the lingual cusps of the mandibular molars, midline, and occlusion are expected to provide considerably better results in actual clinical settings than those in this experiment, if the intermaxillary fixation is actively used. The angle between the mandibular borders, which showed the most variation across individual subjects, was the most sensitive indicator and exhibited a positive correlation with surgical accuracy. Furthermore, it was confirmed to be more accurate for reconstructing the angle in the accurate surgery group. Therefore, the angle is considered a useful index for determining the accuracy of mandibular reconstructive surgery.

One of the limitations of this study was that the data were limited to the results immediately obtained postoperatively. The reason for unavailability of long-term outcome data was that the rabbits tended to die early after mandibulectomy followed by 3D reconstructive surgery in a previous experiment [50]. Feeding difficulties because of stress and pain during surgery are considered the cause of death. Intermaxillary fixation and feeding difficulties because of pain render it difficult to obtain long-term data of mandibular reconstruction in the rabbit model. If intermaxillary fixation can be performed and the feeding difficulties can be resolved using methods such as nasogastric intubation, long-term data in the rabbit model could be obtained. Moreover, constructing a reconstructive surgery model in rabbits for areas unrelated to ingestion or mastication, such as legs, may be an alternative for a more stable research outcome.

This study was based on a 3D customized mandibular reconstruction performed by one surgeon on 20 rabbits. The results immediately obtained postoperatively were extensively analyzed and the causes of error during the surgical stage were assessed from various perspectives. In addition, this study is significant because it proposes various solutions that can provide patients with more accurate clinical outcomes in the future. Furthermore, the errors that occur perioperatively can be reduced if the surgical bone resection guide and implant positioning devices are designed and used as suggested, and if further improvements are made to surgical bone resection guide and implant design. A reconstructive surgery method using a customized 3D implant and surgical bone resection guide will provide more accurate and stable surgical outcomes.

## Conclusions

In this study, reconstructive surgery was performed on a 10-mm mandibular defect model of 20 rabbits aged 50 weeks, using a 3D-customized surgical bone resection guide and a titanium implant. The data collected pre- and postoperatively were analyzed in terms of output accuracy, surgical accuracy, surgical outcome accuracy, correlation between the surgical accuracy and surgical outcome accuracy, and differences in surgical outcomes depending on surgical accuracy. The following results were obtained:

1. The materials used in this study were accurately printed using the 3D printer (SLM machine, model name: SLM 280) with the error range of − 0.03–0.03 mm.
2. The error that occurred during the surgery was within an error range of − 0.4–1.1 mm, which was considerably greater than the error range of the 3D printer.
3. To improve surgical accuracy, it is necessary to devise a positioner to ensure that the bone resection guide is positioned at the correct position as planned.
4. To improve surgical accuracy, it is necessary to design a 3D bone resection guide that would facilitate maintenance of the direction of the bone resection.
5. When designing a bone resection guide, it is necessary to specifically consider the kind of osteotome to be used.
6. For implant stability, the concept of three-point fixation should be applied when the position of fixation is selected to ensure that rotational force would not be applied to the implant.
7. When strong fixation is needed, bilateral cortical bone fixation should be considered in the design.
8. Intermaxillary fixation should be actively used to obtain the positional stability of the implant and maintain occlusal relationship.
9. The angle between the mandibular lower borders is a good indicator for assessing the outcome of mandibular reconstructive surgery.

Future research should be directed toward increasing surgical accuracy. If the aforementioned limitations are addressed, reconstructive surgery of the mandible using a 3D customized surgical bone resection guide and titanium implant may be performed more accurately.

## Data Availability

Not applicable

## Abbreviations

CT: Computed tomography
SLM: Selective laser melting
